# Veterinarians’ Self-Reported Behaviors and Attitudes toward Spectrum of Care Practices

**DOI:** 10.3390/ani14101416

**Published:** 2024-05-09

**Authors:** Emily D. Dolan, Margaret R. Slater

**Affiliations:** Department of Strategy and Research, American Society for the Prevention of Cruelty to Animals, New York, NY 10018, USA; margaret.slater@aspca.org

**Keywords:** spectrum of care, access to veterinary care, incremental care, contextual care

## Abstract

**Simple Summary:**

Access to veterinary care for pet owners is an important part of keeping animals healthy. Not having enough money and other resources can make it hard for pet owners to get veterinary care. Recently, veterinarians have started offering a range of care options to address clients’ needs. But veterinarians still mainly give the most technological and specialized care they learned in their training. The goal of this study was to learn more about veterinarians’ behaviors, knowledge, attitudes, and beliefs about offering more treatments in a range from less to more sophisticated. This study found that many veterinarians do report offering a range, but that it is mostly the veterinarians who feel the most comfortable and confident doing it. When veterinarians had been in practice for more than 20 years, they were less likely to offer a range of services. Rural veterinarians were more likely to offer a range than urban veterinarians. These results offer a reference for more exploration into what veterinarians report about offering a range of care options to people who need help.

**Abstract:**

Access to veterinary care for animal owners is an important part of keeping animals healthy and keeping pets and people together whenever that is appropriate. Insufficient financial and other resources to allocate to veterinary care are major barriers for pet owners to receiving preventative, sick, and emergency services. The veterinary community has begun to incorporate offering a range of diagnostic and treatment options more intentionally in response to clients’ inability to pay and to a lesser extent to mitigate other barriers to care. Many veterinarians are nonetheless oriented toward providing specialized and more sophisticated care based on their training. This study sought to identify the self-reported behaviors, knowledge, attitudes, and beliefs of veterinarians about offering a spectrum of care options (SoC) to clients. The finding that many reported offering SoC is encouraging. However, veterinarians who report comfort and confidence in a variety of aspects of clinical care were most likely to offer SoC. Practitioners in the field for 20 or more years were less likely to offer SoC to clients with financial limitations. Rural veterinarians were more likely to offer SoC to any client compared to urban veterinarians. These results provide a point of reference and potential focus for veterinarians who are not currently offering SoC as well as an exploration of veterinarians’ reported knowledge, behaviors, attitudes, perceptions, and concerns about SoC.

## 1. Introduction

An estimated 164 million dogs and cats live in United States (U.S.) homes, over 24 million of which may be living in poverty [1,2]. Access to veterinary care for owners is an important part of keeping animals healthy and keeping pets and people together. And yet, 25% to 50% of the more than 150 million owned dogs and cats do not receive regular care [3]. A recent study created an index of accessibility for veterinary care and estimated that over 25 million animals were found to live in the least accessible counties of the U.S. [4]. The cost of care has been consistently revealed in recent research as a primary barrier to veterinary care [5,6]. According to the authors of [7], insufficient financial resources to allocate to veterinary care is a major barrier for pet owners. Cost was identified as an overwhelming barrier for all income groups of pet owners, for all types of care (80% for preventative care, 74% for sick care, and 56% for emergency care). In a 2015 study on pet rehoming, respondents with incomes below USD 50,000 were more likely to re-home due to cost [8]. Of those responses, the most common service that could have helped keep their pets was free or low-cost veterinary care (40%). Additionally, those who rehomed due to costswere more likely to surrender to a shelter, increasing the burden on that system. In another study, 80% of pet owners said cost was the reason they were unable to access health care for their pet [7]. Another survey of cat owners in Massachusetts attending a sterilization clinic from 2006 to 2008 revealed that almost 2/3 of cats had never been to a vet, with 44% of those responding the cat was not sterilized because it was too expensive. That study also found an association between income and the number of times a cat had been examined by a veterinarian prior to the utilization of reduced-cost services [9]. While lower-income households are most impacted by the cost of care and have fewer resources and options to overcome other barriers, middle-income households also struggle to access care [10], making this a widespread issue.

While the concept of access to care is multifaceted and can include accessibility and the lack of available services in certain geographic locations, as well as other spatial sociodemographic barriers [11], one important way to define accessible care is care that can be obtained within a client’s means [12]. Because the client is primarily responsible for the full cost of veterinary care and more intervention is often more costly and time consuming, the availability of options for care on a spectrum is a way to reduce the financial burden on clients and improve access to care [6]. A spectrum of care (SoC) includes a variety of diagnostic and treatment options, ranging from technologically advanced and expensive to less advanced and costly [13]. Additional barriers beyond cost for owners include taking time off from work, lack of providers who speak the client’s language, transportation, and geographic distances to providers [3]. Therefore, offering a SoC can be beneficial to meet the needs of animals and their families for a variety of other reasons as well as providing options in line with values and beliefs, conservatively managing risk, and maintaining healthy patient–provider relationships. Providing a spectrum of high-quality services may increase access to needed health care by allowing pet owners to select options that are more feasible yet still provide effective and appropriate care [5,14]. A standard one-size-fits-all approach does not acknowledge the needs and desires of the pet and family [5,14]. While many believe that making available fully or partially subsidized services is the only way to provide more accessible care especially when finances are a barrier, SoC is a separate and distinct way of providing a range of non-judgmental and feasible care options that account for issues reflecting both the veterinarians’ (in their knowledge and skills, availability of services, and recommendations for treatment) along with the patients’ goals, values, culture, and resources [13]. Conceptually, SoC does not necessitate offering subsidized care, potentially making it a more sustainable practice.

Many veterinarians are oriented toward providing the most technologically advanced treatment which is often what is taught in veterinary schools [14,15]. Many recent graduates may not have the knowledge and skillsets to deliver SoC and, therefore, may not feel comfortable offering it. A national study in 2018, however, found that veterinarians overwhelmingly agree pets deserve “some level” of veterinary health care [7]. Therefore, finding ways of increasing access to veterinary care is of paramount importance.

The present cross-sectional study assesses veterinarians’ self-reported behaviors, knowledge, attitudes, and beliefs about offering a spectrum of veterinary care to learn how veterinarians in practice understand SoC and to describe who is and who is not offering it. The study examines respondents’ reports of comfort with aspects of SoC, what veterinarians worry about in relation to SoC, and what they believe veterinary care should be. This can help inform animal health professionals about the status of the veterinary relationship to SoC and help identify new approaches to increase the number of veterinarians providing clients with a SoC. These relationships are examined both when financial limitations are and are not known.

## 2. Materials and Methods

The VIN network represents a group of veterinary professionals, including veterinarians and students, who are able to pay a fee for membership in a community that provides continuing education, assistance with difficult cases, publication access, and other resources to help veterinarians connect with each other and learn about advances in the field. We designed a survey to address the question of veterinary professionals’ attitudes toward offering a spectrum of veterinary care. We assessed the care offered, as well as perceptions and intentions for offering a spectrum of care. The survey was written by a research team and was then refined by the VIN team. The final survey was deployed through an announcement to the VIN network and responses were collected for two weeks beginning on 15 November 2021. The online survey contained a screening question to identify practicing veterinarians from students and non-practicing professionals, information about how long professionals have been practicing, work environment, role, and type of community. To address the primary outcomes, the survey asked questions about how interested professionals were in offering SoC services and their comfort level, agreement with, and experiences with activities around SoC services. The complete survey included items outside of the scope of the present study as it was designed to meet several different needs; see Appendix A for the full survey instrument. 

The characteristics of the survey sample and responses to all questions were described using frequencies and percentages. Analyses were run using Stata/BE 17.0 (StataCorp LP, College Station, TX, USA). A binary variable “owner” was created to describe a practice owner (owner, co-owner, or self-employed) compared to other types of veterinary staff (associate, relief, and contract). A year-in-practice variable by decade was created, as was a variable that grouped states by region (Northeast, Midwest, South, West, Canada, and Other/International). Variables were used that represented whether a provider worked in private practice or other settings and in an urban, suburban, rural, or mixed setting. 

A factor analysis using a polychoric correlation matrix (polychoric, matrix) and promax rotation (factormat, rotate, promax) was conducted to reveal any patterns that could be identified across the large number of items the survey contained that were related to SoC. The strongest factor loading determined which items belonged to each factor. 

To use the items that comprised each factor in a logistic model as categorical variables, Cronbach’s alpha was calculated to ensure the items in each factor had inter-item reliability within a factor. Numerical scores were calculated from the Likert scales by creating a range from “Strongly Disagree” or “Not at All Comfortable” being equal to 1 to “Strongly Agree” or “Extremely Comfortable” being equal to 5. An average score was then calculated for each respondent by taking the mean of the reported scores across the items in each specific factor. Those mean scores for each respondent were then categorized for each factor into 4 categories with roughly equal counts. 

The logistic models used two primary outcomes: “How often do you offer a spectrum of care approach for clients with known financial limitations?” and “How often do you offer a spectrum of care approach for clients with NO known financial limitations?” These items were categorized as often and always compared to occasionally, rarely, and never to create binary outcomes for the logistic models. The logistic models were run using Stata’s logistic command with robust standard errors. Statistically significant main effects were considered with a *p*-value of <0.05, and interactions were explored for potential use in the models using the likelihood ratio test. 

## 3. Results

1160 veterinarians completed the survey. The total number of years since graduation from vet school ranged from 1–57 with a median time of 20 years (Table 1).

Most respondents reported being comfortable or extremely comfortable discussing costs with clients, offering a spectrum of care, suggesting treatment options without diagnosis, and discussing alternatives to advanced options with clients (Table 2). Most respondents agreed or strongly agreed that they regularly offered alternatives to what they felt was best when they became aware of their client’s financial limitations. Most respondents agreed or strongly agreed they felt knowledgeable about lower-cost diagnostic and treatment options for common conditions. Only about half of the respondents, however, agreed or strongly agreed that they considered their client’s circumstances when providing their recommendations, and only about half offered simpler diagnostic tests or treatments first, then more advanced testing or treatment options later if needed.

Approximately 40% of respondents agreed or strongly agreed that professional reputation was an important consideration when they recommended treatment options. Most respondents, however, did not worry that other veterinarians would look upon them unfavorably or that their license would be in jeopardy if they offered a spectrum of care options.

Most respondents agreed or strongly agreed that it was acceptable and the right thing to do to offer a spectrum of care approach to clients, although they also agreed or strongly agreed that it was their responsibility to offer what they think is best for the animal, regardless of the cost to the owners.

### 3.1. Factor Analysis

We identified three main factors from the factor analysis. Factor 1 was labeled as “comfort with SoC” and included the following eight items (Cronbach’s alpha = 0.76): (1) discussing the cost of services with clients; (2) offering a spectrum of care when a client cannot afford what you have recommended; (3) suggesting treatment options without a definitive diagnosis due to a need for limited diagnostic testing; (4) I feel that it is important to remain judgment free when dealing with financially constrained clients; (5) I feel knowledgeable about lower-cost diagnostics and treatment options for common conditions (e.g., skin, ear, GI problems); (6) I feel confident in my ability to effectively communicate a spectrum of care options with clients; (7) I feel it is acceptable for veterinarians to offer a spectrum of care approach to clients; and (8) I believe that offering a spectrum of care is the right thing to do. 

Factor 2 was labeled as “worry about SoC” and contained the following four items (Cronbach’s alpha = 0.66): (1) I am uncomfortable offering anything other than what I think is best for the animal; (2) professional reputation is an important consideration for me when I recommend treatment options; (3) I worry that other veterinarians will look unfavorably on me if I offer a spectrum of care options; and (4) I worry that my license could be jeopardized if I provide a spectrum of care options.

Factor 3 was labeled as “what veterinary care should be” and included the following five items (Cronbach’s alpha = 0.71): (1) people should be able to keep their pets regardless of their ability to afford veterinary care; (2) providing free/discounted care to clients makes it hard for private practice veterinarians to earn a living; (3) I feel it is partly my responsibility to help people obtain veterinary care, even if they can’t afford it; (4) I feel it is acceptable for clients to receive free or discounted veterinary care for their pet without an income screen; and (5) if people can’t afford veterinary care, they shouldn’t have pets. 

### 3.2. Logistic Model: Known Financial Limitations for Client

Logistic regression revealed that when controlling for characteristics of the veterinarian, the odds of offering SoC services to people with known financial limitations increase as veterinarians reported increasing comfort with offering services (Table 3). For example, for the highest grand mean comfort levels of 4.75 to 5, the odds ratio of 5.2 indicates that veterinarians with this highest level of comfort for the questions included in this variable are about 5 times more likely to offer spectrum of care to clients with financial constraints compared to veterinarians with the lowest level of comfort for those questions (see Table 2 for the questions in this variable). When controlling for all other variables, the model shows that the odds of offering SoC services to people with known financial limitations are significantly lower (about 3 to 4 less likely) for 20 years or more in practice compared to 19 years or fewer. 

### 3.3. Logistic Model: Any Client Regardless of Financial Limitations

The logistic regression in Table 4 reveals that controlling for characteristics of the veterinarian, there was a significant increase in the odds of offering SoC as comfort increased compared to the lowest comfort levels. There was a decrease in the odds of offering a SoC approach for clients when financial limitations were not known for veterinarians in urban and suburban settings compared to rural settings. The data show that comfort with offering SoC is the only significant predictor in both statistical models. 

## 4. Discussion

This study is the first to report on a sample of veterinarians’ self-reported behavior, knowledge, attitudes, and beliefs around offering SoC to their clients. The key driver from this dataset was reported as “comfort” with offering a SoC, derived from a combination of eight questions. For clients with known financial limitations, 20 or more years in veterinary practice was also associated with being less likely to offer SoC. For clients without any known financial limitations, being in a rural setting rather than an urban one was associated with a higher likelihood of offering SoC. These findings help identify where and which veterinarians are currently offering SoC, highlight that there are differences based on known financial need, and that there are complex combinations of beliefs, concerns, and skills that drive the behavior of offering SoC. 

This sample of veterinarians from VIN were predominately working in private or corporate practice, as associates in suburban environments. The majority reported high levels of comfort, familiarity, and confidence with SoC, which is what would be anticipated both as socially acceptable responses as well as among a group who is interested in the continuing educational opportunities and collegial discussions and problem solving offered by membership in VIN. Interestingly, and perhaps relatedly, professional reputation was noted as a greater concern for recommending treatment options (40%) than worry about what other veterinarians will think about offering SoC (8%) or licensing concerns (14%). Licensing is anecdotally considered a concern; perhaps it is more about liability and the associated insurance, time, and reputational costs [16,17]. Therefore, it appears that this question about licensing may not be at the heart of liability issues for practicing veterinarians.

Despite very strong support for offering SoC, there was noticeable divergence in responses to questions about whether people should be able to keep their pets regardless of their ability to afford care and providing free or discounted care making it hard to earn a living. These responses indicate some likely unresolved internal conflicts about pet ownership, finances, and veterinary practice. While these questions were included in the factor about “what veterinary care should be” they were not significantly associated with offering SoC. It is possible that these conflicts contribute to the moral stress reported among veterinarians and the reports that limited conversations about costs between clients and veterinarians are occurring [10,14,18,19]. Therefore, fully embracing SoC could decrease moral distress and increase satisfaction with practice by providing a path forward to be able to offer a tailored approach across a continuum of options [5,14].

Alternatively, veterinarians may be conflating SoC and fully or partially subsidized care leading to income concerns. Approximately 60% of veterinarians in the current sample reported agreement that providing free or discounted services makes it hard for private practice veterinarians to earn a living. Regardless, lower-cost or subsidized veterinary care was modeled in computer simulations that support clinics providing low-cost care do not interfere with full-cost clinics as the low-cost services are used by a different sector of patients [20]. Some veterinarians may also believe offering more complex services allows them to maintain their finances and income, perhaps because of outdated knowledge regarding business models. Despite a recognized need for updated and innovative financial models of veterinary care, especially since the COVID-19 pandemic [3,21], there is little research updating economic models of successful veterinary practices. However, expanding accessible care will involve more market segmentation and stratification [3] and ultimately increase access for clients and patients. It may also provide veterinarians with a wider range of practice options, increasing satisfaction and retention in the profession.

In recent years, an increase in emphasis on veterinary and client communication has been evident in the publication of research and in additional training [14,15,22]. Respondents to this survey overwhelmingly disagreed with only offering the best for the animal (88%). At the same time, only slightly more than half considered clients’ circumstances when making recommendations or offering simpler options first. Slightly fewer than half also felt it was partly their responsibility to help people get care when they couldn’t afford it. Clearly, there are still disconnects between beliefs and actions that need to be addressed. Additionally, clients have varied approaches and desires in their communication styles, supporting the crucial role of expert communication for successfully practicing veterinary medicine and meeting client and patient needs [3].

The logistic regression findings that veterinarians in rural practices were more likely to offer SoC to any client regardless of financial need could be linked to better communication and knowledge of their clientele. Perhaps rural veterinarians have had to lean on SoC due to their constraints such as limited options of nearby veterinary clinics for clients (though this study did not determine the availability of alternate resources in any geographic area), being able to tailor their service options to maintain financial viability, clients being familiar or even neighbors, and limited referrals options due to distance so that rural practitioners need to be well versed in all available options.

Because veterinarians report being comfortable with SoC and these data show it is associated with the reported behavior of offering SoC to clients, it is unclear why only about half of the respondents reported considering client circumstances or report offering simpler levels of care before moving on the more complex levels of care. Given that few respondents were concerned with reputation or professional licensing, other barriers must exist around the uptake of offering SoC. It is possible that 20 or more years in practice is a surrogate for some of these unknown barriers. While it might seem like veterinarians would have greater skills and experience in offering a variety of options or in communicating with clients with increased time in practice, other factors such as routines and habits may be a greater influence on whether SoC is offered. External barriers may also exist to managing care for clients, such as practice policies that limit the ability to offer SoC to clients. 

Veterinary students learn a plethora of advanced diagnostics and may make assumptions that the pet owner will want to pursue all options available regardless of priority or cost [22,23,24]. They also may not have practice and confidence in conversations about the relative priority of all options. Further, it is likely that standards of care are misunderstood, and veterinarians may confuse SoC with lower quality practice and not as a continuum of quality care [17].

Studies suggest that service learning and increasing veterinary students’ comfort engaging in behaviors like SoC [23,24,25] is an important way to increase skills and potential comfort with offering SoC as graduate veterinarians. Further, modifying the veterinary curriculum to increase veterinary students’ awareness of the barriers that caregivers face and their knowledge of and comfort with diagnostic and treatment options outside of advanced, “gold-standard” care may have a substantial impact on how students practice once they graduate [22,26].

While most respondents reported feeling that offering SoC was the right thing to do, most also reported that they were obligated to offer what they thought was best. This “best” may be based solely on experience as many common diagnostic and treatment options, including “gold-standard” ones, do not have strong evidence for their efficacy or good data on their success rates. There is a growing field of work being carried out to find evidence for the most effective SoC protocols for a variety of circumstances [23,27,28,29,30] to expand the options and provide reassurance for veterinarians when practicing using SoC options. 

### Limitations

This study had a few important limitations. The first is that we accessed the veterinarians through their VIN membership. This sample is only as representative of veterinarians as that membership. It is likely there are veterinarians who elect not to join VIN and pay the monthly fee who may have different exposure to SoC. Related to this, it is likely that social desirability bias plays a role to some extent in many of the survey responses. Items related to professional reputation, professional repercussions, and doing what is perceived as best in particular, may be vulnerable to bias. With that awareness, the conclusions drawn here are purposefully conservative and framed to reflect participants’ responses as reported. Another limitation of this study is the cross-sectional nature of the data which limits our ability to draw conclusions about the causes of some of the behaviors reported. The data show veterinarians’ self-reported knowledge, attitudes, and beliefs, but little is known at this time about how this connects to the clinical behavior of offering SoC to clients and the self-reports may be overestimated. Further, because of the data collection tool itself, considerations such as differences between private and corporate work settings could not be differentiated. There could be substantial variation based on salary models that this study was not designed to detect. A final limitation was the length of the survey. There were originally multiple purposes for fielding this survey. As a result, shorter length was an important consideration to ensure full participation and reduce drop-out. Therefore, some additional demographic data such as gender were not asked. However, this is the first work of this type, and it does serve as a valuable starting point for future endeavors. 

## 5. Conclusions

The findings here document an important early step in understanding the self-reported behaviors, knowledge, attitudes, and beliefs of veterinarians around offering SoC for their clients. The data offer some of the first insights into who is offering SoC, i.e., those who are most comfortable offering it and therefore, which populations of veterinarians may need more continuing education, examples, or peer support. It is probable that new graduates will be more comfortable with offering SoC given the training and educational emphasis; this is somewhat supported by the finding that being in practice for fewer than 20 years increases the veterinarian’s likelihood of offering SoC. It will be important for future studies to examine what increases comfort, as well as the likely complicated interplay of other factors that may be associated with offering SoC, to understand the link between veterinarians’ comfort offering SoC with their willingness and practical ability to do so. As new evidence for care options across the spectrum gets taken up into the veterinary community, tracking shifts in whether veterinarians become increasingly likely to offer SoC options for clients and patients without sacrificing safety and efficacy will be essential. 

## Figures and Tables

**Table 1:** Demographic information for 1160 veterinarians who responded to the survey in VIN in 2021.

	Freq	Percent
**Years in Practice**		
<10 years	274	23
10–19 years	293	25
20–29 years	290	25
30–39 years	231	20
40 years or more	64	6
**Work Setting**		
Private or corporate practice	1033	89
Animal shelter veterinarian	42	4
Mobile clinic	30	3
Non-profit organization (non-spay/neuter)	26	2
Academia	16	1
Spay/neuter clinic	10	1
Other *	3	<1
**Position**		
Associate	552	52
Owner, co-owner, or self-employed	411	38
Relief or contract	105	10
**Type of Community**		
Urban	213	19
Suburban	545	50
Rural	131	12
Mix of communities	207	19

* The other category contained write-ins for government, resident, and telehealth.

**Table 2:** Frequencies of responses for individual items in a survey of 1160 veterinarians who responded to the survey in VIN in 2021. All items had the full range of responses (1–5) selected by respondents.

Individual Item from Survey	Always or Often Freq (%)	Occasionally, Rarely, or NeverFreq (%)
How often do you offer a spectrum of care approach for clients with known financial limitations? *n* = 1160	1054 (91)	106 (9)
How often do you offer a spectrum of care approach for clients with NO known financial limitations *n* = 1159	712 (61)	447 (39)
	**Not comfortable (1 = not at all and 2)** **Freq (%)**	**Comfortable (3, 4, and 5 = extremely)** **Freq (%)**
Comfort level in discussing the risks and benefits of alternatives to advanced options *n* = 1149	27 (2)	1122 (98)
* Comfort level discussing cost of services with clients *n* = 1139	92 (8)	1047 (92)
* Comfort level in offering SoC when client can’t afford the recommended treatment *n* = 1145	38 (3)	1107 (97)
* Comfort level in suggesting a treatment without a definitive diagnosis *n* = 1148	61 (5)	1087 (95)
	**Strongly disagree, disagree, neutral (1, 2, 3)** **Freq (%)**	**Agree, strongly agree (4, 5)** **Freq (%)**
* I believe that offering SoC is the right thing to do *n* = 1154	132 (11)	1022 (89)
* I feel it is acceptable for veterinarians to offer a spectrum of care approach to clients *n* = 1158	70 (6)	1088 (94)
* I feel that it is important to remain judgement free when dealing with financially constrained clients *n* = 1157	82 (7)	1075 (93)
* I feel confident in my ability to effectively communicate a spectrum of care options with clients *n* = 1157	56 (5)	1101 (95)
* I feel knowledgeable about lower-cost diagnoses and treatments for common conditions (e.g., skin, ear, GI problems) *n* = 1159	33 (3)	1013 (87)
^^^ I am uncomfortable offering anything other than what I think is best for the animal *n* = 1159	1018 (88)	141 (12)
^^^ Professional reputation an important consideration for me when I recommend treatment options *n* = 1155	693 (60)	462 (40)
^^^ I worry that other veterinarians will look unfavorably on me if I offer a spectrum of care options *n*= 1158	1066 (92)	92 (8)
^^^ I worry that my license could be jeopardized if I provide a spectrum of care options *n* = 1159	994 (86)	165 (14)
^&^ People should be able to keep their pets regardless of their ability to afford veterinary care *n* = 1158	816 (70)	342 (30)
^&^ Providing free/discounted care to clients makes it hard for private practice veterinarians to earn a living *n* = 1159	440 (38)	718 (62)
^&^ I feel it is acceptable for clients to receive free or discounted veterinary care for their pet without an income screen *n* = 1158	998 (86)	160 (14)
^&^ If people can’t afford veterinary care, they shouldn’t have pets *n* = 1158	721 (62)	437 (38)
I regularly offer alternatives to what I think is best for the animal when I become aware of clients’ financial limitations *n* = 1158	102 (9)	1056 (92)
I consider client’s circumstances when providing my recommendations *n* = 1158	546 (47)	612 (53)
I generally offer simpler diagnostic tests or treatments first and suggest advanced testing or treatment options later if needed *n* = 1158	564 (49)	594 (51)
I feel it is partly my responsibility to help people get veterinary care even if they cannot afford it *n* = 1159	648 (56)	511 (44)

* Indicates items included in Factor 1 “comfort”; ^^^ indicates items included in Factor 2 “worry”; ^&^ indicates items included in Factor 3 “what veterinary care should be”.

**Table 3:** Logistic regression on how often the veterinarian reports offering a spectrum of care approach for clients with known financial limitations where zero is occasionally, rarely or never and one is often or always. Chi sq: 80.56; pseudo R^2^: 0.12; *n* = 1002.

Variable	Odds Ratio	Robust Standard Error	*p*-Value	95% CI
**Position**				
Owner	0.80	0.22	0.42	0.47–1.37
Associate, contract, or relief, self-employed	Ref.			
**Years in practice**				
<10 years	Ref.			
10–19 years	0.77	0.32	0.54	0.35–1.74
20–29 years	0.35	0.13	0.01	0.17–0.74
30–39 years	0.38	0.15	0.02	0.17–0.83
40 years or more	0.25	0.14	0.01	0.08–0.74
**Work Setting**				
Private or corporate practice	2.78	1.58	0.07	0.92–8.44
Other types of practice (academia, shelter, mobile clinic, non-profit, or spay/neuter clinic)	Ref.			
**Type of community**				
Mix of communities	1.58	0.70	0.30	0.66–3.77
Suburban	1.25	0.42	0.51	0.64–2.43
Urban	0.81	0.32	0.60	0.38–1.75
Rural	Ref.			
**Region**				
West	Ref.			
South	0.86	0.29	0.65	0.45–1.65
Midwest	0.87	0.34	0.72	0.40–1.87
Northeast	0.77	0.28	0.48	0.38–1.58
Canada	0.68	0.31	0.41	0.28–1.67
Other, including international respondents	1.60	0.92	0.41	0.52–4.93
**Comfort with SoC (mean of eight items in the factor)**				
2.3–3.9	Ref.			
4–4.3	1.90	0.54	0.02	1.09–3.31
4.4–4.7	4.69	1.60	<0.001	2.40-9.16
4.8–5	5.21	2.10	<0.001	2.36–11.51
**Worry about SoC (mean of four items in the factor)**				
1.0–1.8	Ref			
2.0–2.3	0.62	0.26	0.26	0.27–1.42
2.4–2.8	0.94	0.43	0.90	0.39–2.28
2.8–4.8	0.46	0.18	0.05	0.21–1.01
**What veterinary care should be (mean of four items in the factor)**				
1.6–2.4	Ref.			
2.6–2.8	0.71	0.31	0.43	0.30–1.66
3.0–3.0	0.90	0.41	0.82	0.37–2.21
3.2–4.4	0.57	0.24	0.18	0.25–1.30

**Table 4:** Logistic regression on how often the veterinarian reported offering a spectrum of care approach for clients with no known financial limitations where zero is occasionally, rarely, or never and one is often or always. Chi sq: 84.01; pseudo R^2^: 0.07; *n* = 1001.

Variable	Odds Ratio	Robust Standard Error	*p*-Value	95% CI
**Position**				
Owner	0.79	0.13	0.15	0.57–1.09
Associate, contract, or relief, self-employed	Ref.			
**Years in practice**				
Less than 10 years	Ref.			
10–19 years	0.90	0.19	0.60	0.59–1.35
20–29 years	0.68	0.14	0.07	0.45–1.03
30–39 years	0.67	0.16	0.08	0.42–1.06
40 years or more	0.54	0.18	0.07	0.28–1.04
**Work Setting**				
Private or corporate practice	0.52	0.22	0.13	0.22–1.21
Other types of practice (academia, shelter, mobile clinic, non-profit, or spay/neuter clinic)	Ref.			
**Type of community**				
Mix of communities	1.10	0.30	0.74	0.64–1.86
Suburban	0.66	0.15	0.06	0.42–1.02
Urban	0.47	0.12	<0.01	0.28–0.79
Rural	Ref.			
**Region**				
West	Ref.			
South	0.72	0.14	0.08	0.49–1.04
Midwest	1.30	0.29	0.25	0.83–2.02
Northeast	1.09	0.24	0.69	0.71–1.68
Canada	1.22	0.34	0.49	0.70–2.11
Other, including international respondents	1.04	0.36	0.91	0.53–2.05
**Comfort with SoC (mean of eight items in the factor)**				
2.3–3.9	Ref.			
4–4.3	1.94	0.39	<0.01	1.31–2.86
4.4–4.7	2.43	0.49	<0.001	1.64–3.61
4.8–5	4.03	0.94	<0.001	2.55–6.37
**Worry about SoC (mean of four items in the factor)**				
1.0–1.8	Ref.			
2.0–2.3	0.93	0.19	0.71	0.61–1.40
2.4–2.8	0.95	0.21	0.81	0.62–1.46
2.8–4.8	0.74	0.16	0.18	0.49–1.14
**What veterinary care should be (mean of four items in the factor)**				
1.6–2.4	Ref.			
2.6–2.8	0.99	0.24	0.97	0.62–1.58
3.0–3.0	1.35	0.34	0.25	0.81–2.22
3.2–4.4	1.24	0.29	0.35	0.79–2.0

## Data Availability

The original contributions presented in the study are included in the article, further inquiries can be directed to the corresponding authors.

## Appendix A

Survey Collection Tool

Veterinarians are vital for maintaining good companion animal welfare as well as recognizing and addressing poor welfare. Because of this, the ASPCA would like to learn more about your approach and attitudes toward several critical aspects of animal welfare. These topics include situations where client finances impose constraints on veterinary care, addressing animal behavior problems, experiences with animal abuse or neglect, and experience providing veterinary care to animals in shelters.

All responses will be confidential, and we will present the summary findings once we have collected sufficient information.

If you have any questions about the survey, please email Mark Rishniw.

(1) What best describes your current situation?

[ ] Practicing in companion animal medicine (small, exotic, equine)

[ ] Student

[ ] None of the above

(2) When did you graduate from Veterinary school?

Drop down menu: 1950–2023

(3) When do you expect to graduate from veterinary school? *

Drop down menu: 2021–2030

(4) What best describes your work environment?

(If you work in more than one setting, select the one where you spend most time)

[ ] Private or corporate practice

[ ] Mobile clinic

[ ] Spay/neuter clinic

[ ] Animal shelter veterinarian (shelter medicine)

[ ] Nonprofit organization (other than spay/neuter clinic)

[ ] Academia

[ ] Other—Write In (Required):

(5) What best describes your position?

[ ] Owner or co-owner

[ ] Associate

[ ] Contract veterinarian

[ ] Relief

[ ] Self-employed

(6) What type of community do you primarily serve in your professional role?

[ ] Urban

[ ] Suburban

[ ] Rural

[ ] Mix of communities

Discounted services

(7) Do you currently offer any free or discounted veterinary services for clients? *

[ ] Yes

[ ] No

(8) To whom do you offer free or discounted veterinary services?

Select all that apply

[ ] To clients who qualify financially through a specific program

[ ] To clients without requiring proof of financial need through a specific program

[ ] To clients who can’t afford the current recommended treatment

[ ] To animal shelter/rescue partner organizations

[ ] To new owners of recently adopted shelter animals

[ ] Friends/family

[ ] Employees

[ ] Other—Write In (Required):

(9) What specific services do you offer at a discount or free?

[ ] Vaccines

[ ] Spay/neuter

[ ] Chronic care management (e.g., diabetes, heart disease, etc.)

[ ] Treatment for basic medical care (e.g., ear infections, skin, eyes, etc.)

[ ] Dentistry

[ ] Treatment for major medical conditions or conditions that require surgery

(10) Please indicate your interest in offering (or having your workplace offer) the following services to select owners for free or at a discount

	**Level of Interest (1 star = No Interest; 5 Stars = Extremely Interested)**
Vaccines	☆ ☆ ☆ ☆ ☆ Not Applicable or don’t know [ ]
Spay/neuter	☆ ☆ ☆ ☆ ☆ Not Applicable or don’t know [ ]
Chronic care management (e.g., diabetes, heart disease, etc.)	☆ ☆ ☆ ☆ ☆ Not Applicable or don’t know [ ]
Treatment for basic medical care (e.g., ear infections, skin, eyes, etc.)	☆ ☆ ☆ ☆ ☆ Not Applicable or don’t know [ ]
Dentistry	☆ ☆ ☆ ☆ ☆ Not Applicable or don’t know [ ]
Treatment for major medical conditions or conditions that require surgery	☆ ☆ ☆ ☆ ☆ Not Applicable or don’t know [ ]

Spectrum of care

For the purposes of this study please use the following definition of spectrum of care: A range of diagnostic and/or treatment options from less technologically advanced/less expensive to more advanced and costly.

(11) Please rate your comfort level with the following activities

	**Level of Comfort (1 Star = Not At All Comfortable, 5 Stars = Extremely Comfortable)**
Discussing the cost of services with clients	☆ ☆ ☆ ☆ ☆ Not Applicable [ ]
Offering a spectrum of care when a client cannot afford what you have recommended	☆ ☆ ☆ ☆ ☆ Not Applicable [ ]
Suggesting treatment options without a definitive diagnosis due to a need for limited diagnostic testing	☆ ☆ ☆ ☆ ☆ Not Applicable [ ]
Discussing risks and benefits of alternatives to more technologically advanced and/or complex options	☆ ☆ ☆ ☆ ☆ Not Applicable [ ]

(12) Please indicate your agreement level to the following statements

	**Strongly Disagree**	**Disagree**	**Neutral**	**Agree**	**Strongly Agree**
I regularly offer alternatives to what I think is best for the animal when I become aware of clients’ financial limitations	[ ]	[ ]	[ ]	[ ]	[ ]
I consider it my responsibility to offer what I think is best for the animal, regardless of cost, to all owners	[ ]	[ ]	[ ]	[ ]	[ ]
I am uncomfortable offering anything other than what I think is best for the animal	[ ]	[ ]	[ ]	[ ]	[ ]
Professional reputation an important consideration for me when I recommend treatment options	[ ]	[ ]	[ ]	[ ]	[ ]
I worry that other veterinarians will look unfavorably on me if I offer a spectrum of care options	[ ]	[ ]	[ ]	[ ]	[ ]
I worry that my license could be jeopardized if I provide a spectrum of care options	[ ]	[ ]	[ ]	[ ]	[ ]
I generally offer simpler diagnostic tests or treatments first and suggest advanced testing or treatment options later if needed	[ ]	[ ]	[ ]	[ ]	[ ]
I consider clients’ circumstances when providing my recommendations	[ ]	[ ]	[ ]	[ ]	[ ]
I feel that it is important to remain judgement free when dealing with financially constrained clients	[ ]	[ ]	[ ]	[ ]	[ ]
I worry that I could get into trouble for offering less than optimal treatment	[ ]	[ ]	[ ]	[ ]	[ ]

(13) Please indicate your agreement level to the following statements

	**Strongly Disagree**	**Disagree**	**Neutral**	**Agree**	**Strongly Agree**
People should be able to keep their pets regardless of their ability to afford veterinary care	[ ]	[ ]	[ ]	[ ]	[ ]
Providing free/discounted care to clients makes it hard for private practice veterinarians to earn a living	[ ]	[ ]	[ ]	[ ]	[ ]
I feel knowledgeable about lower cost diagnostics and treatment options for common conditions (e.g., skin, ear, GI problems)	[ ]	[ ]	[ ]	[ ]	[ ]
I feel confident in my ability to effectively communicate a spectrum of care options with clients	[ ]	[ ]	[ ]	[ ]	[ ]
I feel it is partly my responsibility to help people obtain veterinary care, even if they can’t afford it	[ ]	[ ]	[ ]	[ ]	[ ]
I feel it is acceptable for clients to receive free or discounted veterinary care for their pet without an income screen	[ ]	[ ]	[ ]	[ ]	[ ]
I feel it is acceptable for veterinarians to offer a spectrum of care approach to clients	[ ]	[ ]	[ ]	[ ]	[ ]
I believe that offering a spectrum of care is the right thing to do	[ ]	[ ]	[ ]	[ ]	[ ]
If people can’t afford veterinary care, they shouldn’t have pets	[ ]	[ ]	[ ]	[ ]	[ ]

(14) How often do you offer a spectrum of care approach for clients with known financial limitations?

[ ] Never [ ] Rarely [ ] Occasionally [ ] Often [ ] Always

(15) How often do you offer a spectrum of care approach for clients with NO known financial limitations?

[ ] Never [ ] Rarely [ ] Occasionally [ ] Often [ ] Always
